# Analysis of the *Lactobacillus casei* supragenome and its influence in species evolution and lifestyle adaptation

**DOI:** 10.1186/1471-2164-13-533

**Published:** 2012-10-05

**Authors:** Jeff R Broadbent, Eric C Neeno-Eckwall, Buffy Stahl, Kanokwan Tandee, Hui Cai, Wesley Morovic, Philippe Horvath, Jessie Heidenreich, Nicole T Perna, Rodolphe Barrangou, James L Steele

**Affiliations:** 1 Utah State University Department of Nutrition, Dietetics, and Food Sciences, 8700 Old Main Hill, Logan, UT 84322-8700, USA; 2 University of Wisconsin Biotechnology Center, 425 Henry Mall, Madison, WI, 53706-1580, USA; 3 DuPont Nutrition and Health, 3329 Agriculture Drive, Madison, WI, 53716, USA; 4 University of Wisconsin Department of Food Science, 1605 Linden Drive, Madison, WI, 53706-1565, USA; 5 Present address: PPD Inc, 466 Devon Park Dr, Wayne, PA, 19087, USA; 6 DuPont Nutrition and Health, BP10, F-86220, Dangé-Saint-Romain, France; 7 University of Wisconsin Laboratory of Genetics, 425 Henry Mall, Madison, WI, 53706-1580, USA

**Keywords:** *Lactobacillus casei*, Lactic acid bacteria, Comparative genomics, Pan-genome, Supragenome, Evolution, Adaptation

## Abstract

**Background:**

The broad ecological distribution of *L. casei* makes it an insightful subject for research on genome evolution and lifestyle adaptation. To explore evolutionary mechanisms that determine genomic diversity of *L. casei*, we performed comparative analysis of 17 *L*. *casei* genomes representing strains collected from dairy, plant, and human sources.

**Results:**

Differences in *L*. *casei* genome inventory revealed an open pan-genome comprised of 1,715 core and 4,220 accessory genes. Extrapolation of pan-genome data indicates *L*. *casei* has a supragenome approximately 3.2 times larger than the average genome of individual strains. Evidence suggests horizontal gene transfer from other bacterial species, particularly lactobacilli, has been important in adaptation of *L*. *casei* to new habitats and lifestyles, but evolution of dairy niche specialists also appears to involve gene decay.

**Conclusions:**

Genome diversity in *L*. *casei* has evolved through gene acquisition and decay. Acquisition of foreign genomic islands likely confers a fitness benefit in specific habitats, notably plant-associated niches. Loss of unnecessary ancestral traits in strains collected from bacterial-ripened cheeses supports the hypothesis that gene decay contributes to enhanced fitness in that niche. This study gives the first evidence for a *L*. *casei* supragenome and provides valuable insights into mechanisms for genome evolution and lifestyle adaptation of this ecologically flexible and industrially important lactic acid bacterium. Additionally, our data confirm the Distributed Genome Hypothesis extends to non-pathogenic, ecologically flexible species like *L*. *casei*.

## Background

Lactic acid bacteria (LAB) constitute a group of Gram-positive, non-sporulating, nutritionally fastidious, and strictly fermentative bacteria that produce lactic acid as the major end product from carbohydrate [1]. *Lactobacillus*, which currently holds 177 species http://www.bacterio.cict.fr/, is by far the largest and most diverse genus of LAB. Different species of lactobacilli are important components of the oral cavity as well as the gastrointestinal and reproductive tracts of vertebrates, while others are indigenous to milk, plant material, and meat environments [2]. Many play important roles in both traditional and commercial-scale food and feed fermentations, or food spoilage.

A few species of *Lactobacillus* show remarkable ecological adaptability and may be recovered from a variety of diverse habitats [2]. *Lactobacillus casei*, for example, has been isolated from raw and fermented dairy (especially cheese, where they often emerge as the dominant adventitious or “nonstarter” species during ripening [3]) and plant materials (e.g., wine, silage, pickles, and kimchi), as well as the oral cavity and gastrointestinal tracts of humans and animals [2]. As an aside, debate over the taxonomy of *L*. *casei* in recent years has led to interchangeable use of *L*. *casei* and *L*. *paracasei* in the literature. All of the strains included in this study show >99% identity to the 16S rRNA sequence of *L*. *casei* ATCC 334, which is the current type strain for *L*. *paracasei*[4].

The broad ecological distribution of *L*. *casei* reflects a metabolic flexibility that has fueled widespread application of the species in the food and health industries; different strains are employed as acid-producing starter cultures for milk fermentation, as adjunct cultures to accelerate or intensify flavor development in bacterial-ripened cheeses, and as probiotics to enhance human or animal health [5-7]. The Food and Agriculture Organization and the World Health Organization of the United Nations define probiotics as “live microorganisms which when administered in adequate amounts confer a health benefit on the host” [8]. Strains used as starter cultures or probiotics must, of course, be nonpathogenic to humans even when consumed in very high numbers (e.g., 10^9^-10^10^ cfu/dose). Thus, the wide ecological distribution of *L*. *casei* makes this species a particularly interesting and relevant subject for research on genetic diversity, genome evolution, and lifestyle adaptation.

Bacterial genome evolution and adaptation are thought to occur via three major processes: 1) modification of existing genes by mutation with vertical inheritance [9-12]; 2) acquisition of exogenous genes or gene clusters by bacteria through horizontal gene transfer (HGT) that impart a fitness benefit [13-17]; and 3) deletion or decay of genes that no longer confer a fitness benefit [18-20]. Comparative genome analysis of lactobacilli and other LAB has shown that all three mechanisms have contributed to the adaptation of these microbes to new habitats and lifestyles, but gene decay and acquisition by HGT appear to be especially dominant forces [21-28]. In *L*. *casei* and many other LAB, genome evolution is expected to reflect adaptation of the species to dynamic, nutritionally variable and ancient environments, such as the gastrointestinal tract and plant materials, as well as relatively recent expansion into a more constant and nutrient-rich milk-based niche. Not surprisingly, comparative genome analysis between *L*. *casei* ATCC 334 and other sequenced lactobacilli confirmed *L*. *casei* has features, including a relatively high number of carbohydrate-related genes and IS elements [21,28], which are consistent with a metabolically and genetically versatile lifestyle [28,29].

The evolutionary history of *L*. *casei* has been viewed through its population structure; multi-locus sequence typing (MLST) of 40 *L*. *casei* strains isolated from different niches indicated the species had diverged into three lineages [30]. Separation of two major clusters occurred approximately 1.5 million years ago; one large cluster contained strains recovered from human, plant, and dairy sources, while the second and smaller cluster was predominantly comprised of corn silage isolates. In contrast, the third major cluster included 15 strains that had been isolated from cheese in the United States, Australia, and Europe, and was predicted to reflect recent divergence of strains that were highly specialized for this relatively new ecological niche (manufacture of cheese is thought to have begun ~8,000 years ago) [30,31]. This cluster of cheese specialists was also revealed in a follow up study that explored *L*. *casei* genetic diversity by comparative genome hybridization (CGH) of 21 strains against an ATCC 334 whole genome microarray [28]. That work further suggested that adaptation to the cheese environment had been accompanied by extensive decay of genes involved in carbohydrate utilization and transcriptional regulation [28]. Metabolic simplification via the loss of genes for carbohydrate metabolism has been associated with adaptation of other LAB species to the nutrient-rich milk environment [32-35]. Intriguingly, many of the genes that *L*. *casei* cheese specialists lacked were associated with genomic islands postulated to function in lifestyle adaptation of other *L*. *casei* strains [28].

CGH data for the 21 strains as well as comparative genome analysis between strains ATCC 334 and BL23 identified numerous hypervariable regions and genomic islands, respectively, some of which showed an atypical base composition that is commonly associated with gene acquisition by HGT [28]. The diversity and distribution of these regions among different strains indicated that: i) there was a large pool of accessory genes in the population; ii) most *L*. *casei* strains were likely niche generalists that could exploit a variety of habitats and tolerate a wide range of environmental conditions; and iii) HGT has played a significant role in the evolution, lifestyle adaptation, and metabolic diversity of *L*. *casei*[28]. However, these conclusions were softened by the knowledge that microarray-based CGH analysis cannot identify genes that may be present in the test strain but absent from the reference strain, and because the ecological origin of strain BL23 is unclear [36].

In recent years, the availability of multiple genome sequences for a single species has demonstrated that a number of pathogenic bacteria possess an extensive pan-genome or “supragenome” that may be several fold larger than the genome of any single strain [22,23,25,37-40]. This discovery has given rise to the Distributed Genome Hypothesis (DGH), which postulates that access to a supragenome through homologous recombination allows individual strains to rapidly shuffle their genetic information and overcome host defense responses [41]. Although the DGH has been almost exclusively applied to describe genome evolution in pathogens, the circumstances upon which it is founded (i.e., cells aggregate into polyclonal or polymicrobial biofilms which facilitates HGT) [41] are widespread in the microbial world. Baumdicker et al. [42] recently showed the DGH extended to nonpathogenic bacteria, and it was our hypothesis that a distributed supragenome would drive genome evolution and lifestyle adaptation in an ecologically flexible species such as *L*. *casei*.

To test this hypothesis, we collected draft sequences for 12 strains that had been isolated from dairy (n = 5), plant (n = 5), or human (n = 2) sources that appear to provide broad representation for the genetic diversity in *L*. *casei*[28,30], and performed comparative genome analysis between these strains plus the complete sequences from 5 additional *L*. *casei* strains (ATCC 334, BL23, Zhang, BD-II, and LC2W) in the public database [21,43-46].

## Results and discussion

### *L*. *casei* genome features

Genome features of the 17 *L*. *casei* strains included in the study, which provide a broad representation of genetic, ecological, and geographical diversity in the species, are presented in Tables 1 and 2. The 12 new draft sequences had a range of coverage between 17X and 133X (mean = 31.6X) and ranged in total contig number from 28–167 (mean = 99 contigs) (Table 1). Using a set of only three closed genome references (ATCC 334, BL23, and Zhang), we were able to determine orientation and order the contigs of each draft. The contig numbers listed in Table 1 were obtained after contigs were ordered and oriented in Mauve and the draft was aligned end-to-end based on the advanced order, which reduced the number of contigs obtained from the *de novo* assembly by an average of 20% (data not shown). Each of the genomes was assembled into scaffolds based on the placed and unplaced contigs from Mauve, with the unplaced contigs being ordered by name at the end of the scaffold.

**Table 1:** **Genome features of the 17 *L*. *casei *strains used in the study**^**1**^

**Strain**	**Origin**	**Ave. coverage**	**Number contigs**	**Total bp in contigs**	**% GC**	**Plasmid DNA**	**GenBank accession number**	**CDS features**	**tRNAs**	**Reference or source**
ATCC 334	Swiss cheese	8X	2	2,924,325	46.6	1	NC_008526 and NC_008502	2,643	59	[21]
M36	Cheddar cheese	19X	78	3,152,126	46.3		AFYO00000000	3,001	57	[30]
UW1	Cheddar cheese	21X	143	2,865,538	46.4		AFYR00000000	2,826	54	[30]
UW4	Cheddar cheese	22X	122	2,758,298	46.4		AFYS00000000	2,689	57	[30]
Zhang	Koumiss	6X	2	2,898,335	46.4	1	NC_014334 and NC_011352	2,723	59	[44]
BD-II	Koumiss	381X	2	3,127,288	46.3	1	CP002618 and CP002619	3,069	59	[45]
LC2W	Dairy product	98X	2	3,077,434	46.3	1	CP002616 and CP002617	3,019	58	[46]
Lc-10	Dairy product	24X	76	2,951,397	46.4		AFYT00000000	2,780	58	This study
Lpc-37	Dairy product	133X	150	3,075,253	46.3		AFYU00000000	2,861	58	This study
BL23	Unknown	34X	1	3,079,196	46.3	0	NC_010999	2,977	60	[43]
12A	Corn silage	23X	28	2,885,619	46.4		AFYJ00000000	2,702	57	[30]
21/1	Corn silage	26X	75	3,215,878	46.2		AFYK00000000	3,080	57	[30]
32G	Corn silage	17X	42	3,011,496	46.4		AFYL00000000	2,920	57	[30]
A2-362	Wine	24X	167	3,361,266	46.1		AFYM00000000	3,262	58	[28]
UCD174	Wine	25X	116	3,071,637	46.4		AFYQ00000000	3,020	57	[28]
T71499	Human blood	30X	55	3,000,122	46.2		AFYP00000000	2,796	57	[30]
CRF28	Human blood	24X	57	3,036,548	46.3		AFYN00000000	2,911	54	[30]

^1^Determined for all genomes using the ASAP database (https://asap.ahabs.wisc.edu).

**Table 2:** **Orthologous clusters in the *L*. *casei *supragenome**^**1**^

**Strain**	**Orthologous clusters**						
	**Total**	**Core**	**Pan**	**Distributed**	**Unique**	**% Non-core**	**% Unique**
ATCC 334	2,511			796	54	32	2
M36	2,918			1,203	91	41	3
UW1	2,700	1,715	5,935	985	203	36	8
UW4	2,593			878	187	34	7
Zhang	2,649			934	49	35	2
BD-II	2,936			1,221	19	42	1
BL23	2,866			1,151	54	40	2
LC2W	2,893			1,178	29	41	1
Lc-10	2,715			1,000	104	37	4
Lpc-37	2,799			1,084	37	39	1
12A	2,651			936	47	35	2
21/1	2,958			1,243	183	42	6
32G	2,821			1,106	174	39	6
A2-362	3,108			1,393	326	45	10
UCD174	2,879			1,164	240	40	8
T71499	2,745			1,030	85	38	3
CRF28	2,851			1,136	148	40	5

^1^Downloaded from ASAP (http://www.asap.ahabs.wisc.edu) September 19, 2011.

Although the final contig order was not independently validated for all 12 draft genomes, an optical restriction map [47] was used as a reference to validate assembly of the Lpc-37 genome by this approach (see Additional file 1: Figure S1 in supplementary online material). The overall arrangement of the contig order for Lpc-37 was done using progressive Mauve, and alignment of the contigs to the optical map demonstrates that the method of assembly and ordering yielded a well ordered draft, with only a few major gaps. The optical map gave an estimated genome size of 3,014,302 bp with a total concatenated ordered draft length of 2,916,119 bp (~97% coverage). Based on the alignment between optical map and draft assembly, the size difference is likely derived from 3 major sequence gaps approximately 30, 70, and 10kb in size (Additional file 1: Figure S1).

**Figure 1:**
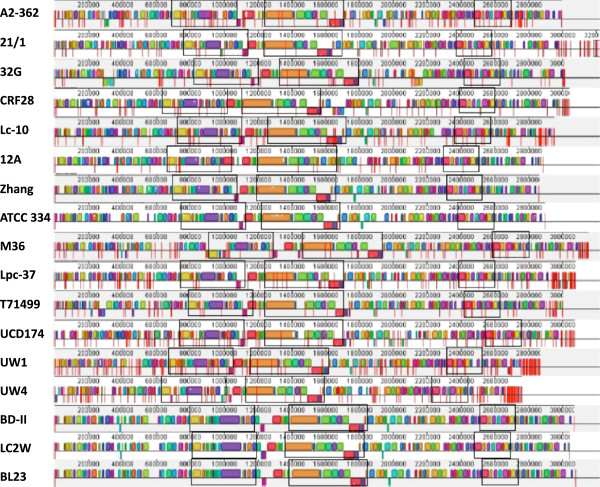
**Alignment of *L*. *casei *genomes.** Five closed and 12 new draft genomes were aligned using progressive Mauve. Three boxed areas outline large highly collinear regions where the order of Locally Collinear Blocks is highly conserved among all 17 strains. The boxes are a fixed length, so that all region size can be compared, and maps were adjusted to the left to reflect length differences. Vertical red lines indicate contig boundaries, and unplaced contigs were sorted to the right end of each map.

The number of predicted CDS features in each genome ranged from 2,643 to 3,262, with an overall GC content of 46.1-46.6% (Table 1). Comparative genomics of the resulting architecture revealed synteny was relatively high across the *L*. *casei* genomes, with several large blocks of highly conserved gene content across all 17 *L*. *casei* strains (Figure 1). These data also indicate that genome size differences were not due to major chromosomal insertions, deletions or re-arrangements.

**Figure 2:**
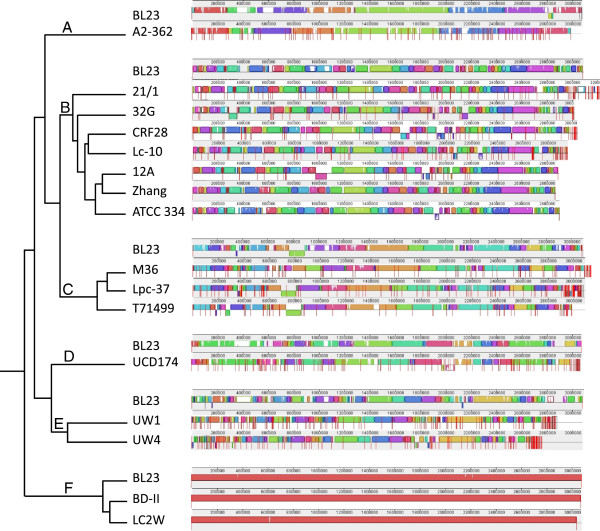
**Hierarchical clustering of 17 *L*. *casei *strains based on overall gene content. **Members of each cluster were aligned using Mauve, and vertical red lines indicate contig boundaries. Locally Collinear Blocks (LCBs) are colored to reveal harmonization within each cluster, but do not have identity to LCBs of the same color outside a particular cluster. The *Lactobacillus casei *BL23 genome was used as a reference to order and orientate contigs for strains included in all clusters except B, where strains 21/1, 32G, and Lc-10 were ordered using the *L*. *casei *Zhang genome as a reference, and strains CRF28 and 12A were ordered based on *L*. *casei *ATCC 334. The BL23 alignment is shown with cluster B to compare genomic similarity.

Sequence homology was determined even more accurately when strains were aligned based on gene content (Figure 2), so that most of the diversity is observed as indels. As expected, the number of Locally Collinear Blocks (LCBs) detected using the progressive Mauve alignment increased with the number of genomes compared. Hierarchical clustering of strains based on overall gene content yielded a dendrogram with 6 clusters, designated A-F (Figure 2). When members of each cluster were aligned to each other or to their closest reference genome, the number of LCBs was reduced, and the overall sequence homology detected between the strains was apparent based on the height of the similarity profile within each LCB (Figure 2). No major genomic rearrangements were detected within any of the draft sequences. Content conservation was even higher within clusters (Figure 2), most notably for clusters E and F. Even within clusters B and C, which contain 7 and 3 strains, respectively, overall synteny was high with mostly localized polymorphic content (Figure 2).

**Figure 3:**
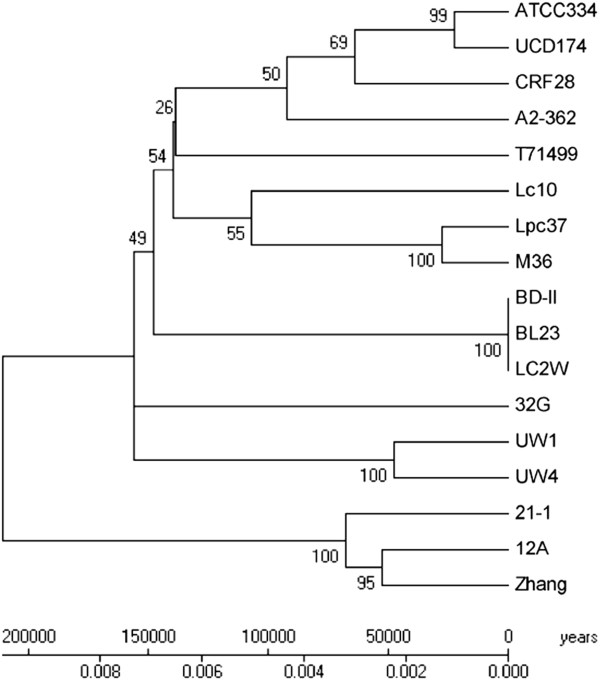
**Genetic relationships among 17 *L*. *casei *strains as defined by multilocus sequence typing. **Concatenated sequences of 6 MLST loci (*ftsZ*, *metRS*, *mutL*, *pgm*, *nrdD*, and *polA*) were analyzed as described previously [30].

Comparison of the gene content-based dendrogram (Figure 2) to an MLST-based phylogenetic tree for the 17 *L*. *casei* strains examined in this study (Figure 3) revealed similar clustering for strains in gene content clusters A, E, F, and parts of B and C. However, MLST-derived relationships among the remaining strains did not resemble those derived from overall gene content. The basis for this observation is well understood; MLST-based phylogeny reflects relatively slow genome evolution caused by point mutations and selective pressure, whereas the gene content dendrogram captures more rapid (and unpredictable) large-scale insertion and deletion events. Thus, variations between gene content- and MLST-based dendrograms are expected in bacterial species like *L*. *casei* that display frequent intra-species recombination [22,25,28,30].

### Characterization of the *L*. *casei* core and supragenome

**Figure 4:**
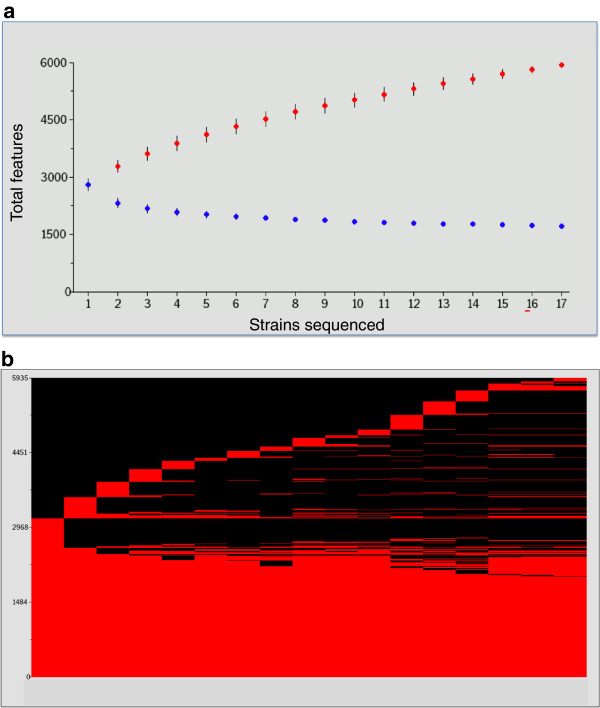
**Feature numbers for the core and pan-genome across 17 *L*. *casei *strains.** Panel **A **shows numbers of total features in the core (blue) and pan-(red) genome as a function of the number of strains sequenced. The average of 500 random permutations of the genome order is presented for the pan and core genome content; the error bars represent the standard deviation of these results. The heat map in panel **B **illustrates the distribution of core and accessory genes (combined in red) across the 17 sequenced strains. New accessory genes that are contributed to the pan-genome by each strain's sequence are depicted as a red cap at the top of each strain name. Black regions under that cap indicate features missing in that strain but present in one or more of the other sequenced *L*. *casei*.

Comparative genome analysis between the five complete and 12 draft genomes provided new insights to the genetic diversity of *L*. *casei*. The *L*. *casei* genome encodes an average of 2,800 (±151) orthologous clusters, of which 1,715 were common to all 17 strains analyzed (“core genome”). Graphing the numbers of core and total features ("pan-genome", or total number of different genes found within a species) as a function of the number of strains sequenced revealed the slope for core gene number was approaching an asymptote, whereas the pan-genome continued to expand even after compilation of 17 genomes (Figure 4A). Overall, differences in *L*. *casei* genome inventory reveal an open pan-genome comprised of 1,715 core and 4,220 accessory orthologous clusters identified to date, including a large number of unique orthologous clusters in each strain (range = 19 to 326, with an average of 119) (Table 2 and Figure 4B). Extrapolation of these data using the binomial mixture model described by Snipen et al. [48] yields an estimated core genome of 1,600 orthologous clusters and a pan-genome of 9,072 total orthologous clusters. These findings indicate *L*. *casei* has a supragenome that is approximately 3.2 times larger than the average genome of individual strains, and support the hypothesis that the relative size and content of the pan-genome is an indicator of genetic plasticity and potential for environmental adaptation within a species [48].

### Strain-specific features of the *L*. *casei* supragenome

Protein homology searches with strain-specific accessory gene products (i.e., unique to a particular strain) revealed 8-87% (mean = 54%) of these proteins had highest similarity to gene products found in other strains of *L*. *casei* or *Lactobacillus paracasei* (see Additional file 2: Table S1 in the online supplementary material). The lowest fractions (<50%) were found in the two wine isolates (A2-362 and UCD174) and three dairy strains (Lc-10, ATCC 334, and Zhang). Expansion of the analysis to include gene products with greatest homology to proteins from other species of lactobacilli accounted for 45-95% (mean = 77%) of the strain-specific accessory genes products. For example, different proteins with very high amino acid identity (E value < 1e^-80^) to orthologs in the closely related species *Lactobacillus rhamnosus* were found in all *L*. *casei* strains except three dairy isolates (M36, BD-II, and BL23). Additionally, strain-specific accessory genes encoding orthologs with high homology to proteins from a broad range of other *Lactobacillus* species including *L*. *plantarum*, *L*. *fermentum*, *L*. *brevis*, *L*. *buchneri*, *L*. *coryniformis*, *L*. *coleohominis*, *L*. *farciminis*, *L*. *helveticus*, *L*. *hilgardii*, *L*. *jensenii*, *L*. *kefiranofaciens*, *L*. *ois*, *L*. *pentosus*, and *L*. *salivarius* were sporadically distributed among the 17 *L*. *casei* strains examined (Additional file 2: Table S1). Many of these lactobacilli are only distantly related to *L*. *casei*, but all share at least one ecological niche with this species and theoretically might have contributed to the diversity of the *L*. *casei* supragenome. There were, for example, clear relationships between ecological co-localization with particular *Lactobacillus* species and the unique accessory gene content of individual *L*. *casei* strains. *L*. *coryniformis*, for example, is commonly found in fermented plant material (e.g., silage) [2] and accessory gene products with very high homology scores to various proteins from this species were found within each of the five silage and wine isolates, but not in any of the human or dairy strains (Additional file 2: Table S1). Similarly, *L*. *casei* strains of dairy origin had a greater prevalence of accessory genes whose products gave very high homology scores to different proteins from *L*. *fermentum* (Additional file 2: Table S1), a species commonly found in milk products [2].

Overall, *L*. *casei* plant isolates showed the most diverse repertoire of strain-specific accessory genes. Genes encoding orthologs with high amino acid identity to different proteins from *L*. *plantarum*, *L*. *buchneri*, and *L*. *pentosus*, for example, were most prevalent in plant isolates, as were proteins with orthologs in distantly related bacteria such as *Enterococcus*, *Streptococcus*, *Bacillus*, and *Clostridium* species (Additional file 2: Table S1). Finally, accessory genes with very high homology scores to various proteins from *Listeria* sp. were detected in each of the human *L*. *casei* isolates and in the three dairy strains, but not in any of the plant isolates, even though *Listeria* are commonly recovered from vegetation [49].

If niche-associated gene exchange contributes to the composite nature of the *L*. *casei* supragenome, and this process is important to *L*. *casei* strain evolution and lifestyle adaptation, then one might expect to find evidence of relatively recent events among different strains. Indeed, several insertion sequence elements in *L*. *casei* strains shared at least 99% nucleotide sequence identity with elements that have been identified in the ge nomes of *L*. *rhamnosus*, *L*. *brevis*, *L*. *buchneri*, *L*. *fermentum*, *Oenococcus oeni* and even *Listeria innocua* (see Additional file 3: Table S2 in the online supplementary material). More interestingly, *L*. *casei* strains UCD174, BD-II, and UW1 each possess a unique polycistronic region encoding features associated with lifestyle adaptation [28] that share very high (98-99%) nucleotide sequence identity with genomic regions in *L*. *plantarum* or *L*. *brevis* (see Additional file 3: Table S2 and Additional file 4: Figure S2 in the supplementary online material). Only one strain of *L*. *brevis* has been sequenced to date, but nucleotide BLAST searches showed each of the clusters with homology to *L*. *plantarum* was common among sequenced strains of that species.

The four-gene cluster found in *L*. *casei* UW1 is virtually identical to a locus in *L*. *brevis* ATCC 367 that encodes an ABC sugar transport system of unknown specificity (Additional file 4: Figure S2A). Tests on subset of nine stains, including UW1, for the ability to grow in CDAA supplemented with one of 60 different substrates did not reveal any capability that was unique to strain UW1 (see Additional file 5: Figure S3 in supplementary online material), so the function of this gene cluster remains unclear. In both species, the cluster is flanked on one side by a gene for transposase (Additional file 4: Figure S2A), and the G+C content of the cluster (ORFs range from 37-39%) is considerably lower than that of either species' genome (46%) (Table 1 and Additional file 3: Table S2), suggesting the cluster may have been acquired by one or both species from a third donor.

The polycistronic cluster identified in *L*. *casei* BD-II is plasmid borne, and carries five of the six genes that comprise the *L*. *plantarum lar* operon [50] (Additional file 4: Figure S2B). Goffin and coworkers [50] showed *lar* is required for lactate racemization activity in *L*. *plantarum*, but the function of most *lar*-encoded proteins, or even whether all six genes are required for this activity, remains unknown. D-lactate is a component of the cell wall in *L*. *plantarum* and *L*. *casei*, and Goffin et al. [50] suggested Lar activity may provide the cell with a rescue pathway for D-lactate production under conditions that inhibit D-lactate dehydrogenase activity. The *lar* genes of *L*. *casei* BD-II show 98-99% nucleotide sequence identity to their counterparts in *L*. *plantarum*, and the BD-II locus is flanked on each side by transposase genes (Additional file 4: Figure S2B). There are no transposase genes in the immediate vicinity of the *lar* operon in *L*. *plantarum*. Collectively, these observations provide good evidence that the *lar* locus in *L*. *casei* BD-II was acquired via HGT from *L*. *plantarum*, but its role, if any, in lifestyle adaptation must yet be determined.

The third and most compelling example of lifestyle evolution via HGT was found in the wine isolate *L*. *casei* UCD174. This bacterium contains a polycistronic cluster for L(+)-tartrate catabolism and malate transport that was previously thought to be one of three defined *L*. *plantarum*-specific gene clusters [51]. Tartaric and malic acids are the primary acids in grapes and therefore the strongest acids in wine. Tartrate dehydratase allows cells to convert tartrate to oxaloacetic acid, an important metabolic intermediate, and malate transport and metabolism are known to enhance acid tolerance in *L*. *casei* and other lactic acid bacteria [52-54]. Thus, acquisition of this cluster by UCD174 very likely promoted survival and adaptation of this strain to the acid environment of wine. Features of the UCD174 tartrate dehydratase cluster, including 99% nucleotide sequence identity over the cluster and associated *aroAB* genes with the corresponding *L*. *plantarum* locus, and the presence of flanking transposase genes in UCD174 (Additional file 4: Figure S2C), provide strong evidence that the locus was acquired by HGT from *L*. *plantarum*.

Finally, we have noted *L*. *rhamnosus* might be an important source of genetic diversity in *L*. *casei*, but also found evidence that the reverse may be true. Of the nine publicly available complete or draft genome sequences for *L*. *rhamnosus*, only two strains, GG (ATCC 53103) and LMS2-1, contain a genomic region that encodes three secreted LPXTG-like pilin proteins (SpaCBA) plus a dedicated sortase for their export [55]. Functional genomic studies showed the SpaCBA pilus promotes adhesion to intestinal epithelial cells, and may function to modulate interleukin-8 expression that is induced by lipoteichoic acid or other surface molecules [56]. While the *spaCBA* locus and associated sortase are clearly uncommon among *L*. *rhamnosus* strains, it is fully conserved in *L*. *casei* strains ATCC 334, BL23, Zhang, and T71499, and present but probably inactive (due to frameshifts or deletions) in *L*. *casei* strains 21/1, M36, CRF28, UW4, A2-362, 32G, Lc-10, and Lpc-37. Collectively, these strains span the major MLST-defined genetic lineages for *L*. *casei*[28,30], which suggests the genes were not recently acquired. The *spaCBA* and sortase genes of *L*. *casei* ATCC 334 show 95-99% nucleotide sequence identity to their counterparts in *L*. *rhamnosus*, whose locus is also flanked by transposase genes that are virtually identical to elements found in *L*. *casei*. There are no transposase genes in the immediate vicinity of the *spaCBA* cluster in the *L*. *casei* strains sequenced to date. Collectively, these observations provide compelling evidence that the *spaCBA* locus in *L*. *rhamnosus* GG and LMS2-1 may have originally been acquired via HGT from *L*. *casei*.

Overall, the composite nature of the *L*. *casei* strain-specific accessory gene pool and the presence of gene clusters in some strains that appear to have been recently acquired support our hypothesis that evolution of the *L*. *casei* supragenome has been heavily influenced by ecological co-localization with other bacterial species. Placed within the greater context of the DGH, we propose that *L*. *casei*, and probably other ecologically flexible species, have access to a supragenome whose composition is not exclusive to the species, but instead might be viewed as a subset of the microbial metagenome within a particular niche. While the primary mechanism(s) for supragenome access by *L*. *casei* are unknown, natural transformation has never been demonstrated in lactobacilli, and the prevalence of IS elements and plasmid-linked traits among genes that appear to have been recently acquired (see Additional file 3: Table S2) suggest that conjugation may be a key driver of HGT in *L*. *casei*. However, the widespread distribution of phage-related proteins among the *L*. *casei* accessory gene pool (Additional file 2: Table S1) suggest transduction could also be an important mechanism for genome evolution in this species.

### Adaptive immunity against invasive DNA

Although conjugation and transduction may be important mechanisms for HGT in *L*. *casei*, these and other bacteria have also acquired sophisticated mechanisms to combat invasive bacteriophage and plasmid DNA. One key example is the CRISPR-Cas adaptive immunity system, which consists of clustered regularly interspaced short palindromic repeats (CRISPR) adjacent to *cas* (CRISPR-associated) genes. The CRISPR loci are comprised of partially palindromic repeats separated by short stretches of "spacer" DNA that are acquired from invasive bacteriophage or plasmid DNA. Once present, these spacer sequences allow cells to recognize and cleave invasive DNA that contains those sequences [57-60]. Two distinct types of CRISPR loci were identified in the *L*. *casei* genomes. These two loci are typically characterized by idiosyncratic CRISPR repeats: 5’-GTCTCAGGTAGATGTCGAATCAATCAGTTCAAGAGC-3’ for the Type II-A (Lsal1 family) locus, and 5’-GTTTTCCCCGCACATGCGGGGGTGATCCC-3’ for the Type I-E (Ldbu1 family) locus. Such repeats have been previously identified in a variety of lactobacilli, including *L*. *salivarius*, *L*. *casei* and *L*. *rhamnosus* for Lsal1, and *L*. *casei*, *L*. *delbrueckii*, *L*. *fermentum*, *L*. *acidophilus* and *L*. *brevis* for Ldbu1 [61]. Overall, CRISPR repeats were highly conserved, with >97% typical repeats across Lsal1, and >96% typical repeats for Ldbu1 (data not shown).

**Figure 5:**
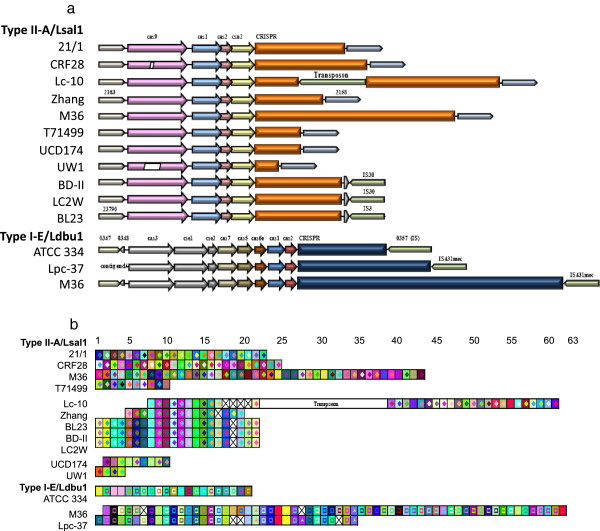
**Graphic representation of CRISPR elements in *L*. *casei *genomes.** Panel **A**, graphic representation of CRISPR-*cas *loci. Top, Type II-A CRISPR-Cas systems; Bottom, Type I-E CRISPR-Cas systems. *cas *genes are represented by colored arrows, while CRISPR repeat-spacer arrays are represented as orange (top) and blue (bottom) boxes. IS elements are colored in green. Numbered genes represented by narrow box arrows refer to previously published nomenclature. Homologous genes are represented using identical colors. Hashed elements represent gaps in the genome draft sequence. Panel **B**, graphic representation of CRISPR spacers across the two CRISPR-Cas types found in *L*. *casei*. Each unique spacer sequence is represented as a specific combination of two colors. Repeats are not included. Missing spacers are represented by crossed squares.

Lsal1-type CRISPR loci were identified in 11 strains, while Ldbu1-type loci were identified in 3 strains, with both types present in the M36 genome (Figure 5A). Only four strains (32G, A2-362, 12A, and UW4) did not possess CRISPR loci. The *cas* content for the Lsal1 loci is typical of Type II-A systems [62], with the universal *cas1* and *cas2*, in combination with the *cas9* signature gene. Also, a tracrRNA homologous to those found in Type II systems [63] was identified in the intergenic region between *cas9* and *cas1*. Though universal genes were highly conserved, up to 19% nucleotide polymorphism was observed for the *cas9* signature gene. The genomic location of these loci across the 13 strains was consistent, and occurred immediately following LCABL_23790 homologs (Figure 5A). The *cas* content for Ldbu1 is typical of Type I-E systems [62], with the universal *cas1* and *cas2*, in combination with the *cas3* signature gene, and Cascade-encoding genes, notably *cas6e*[62,64].

As expected, the spacer content was hypervariable across CRISPR loci from different strains, with spacer numbers ranging from 4 to 44 for Lsal1, and between 21 and 60 for Ldbu1 (Figure 5B). Of note, identical sets of spacers were conserved across cluster F strains (BL23, BD-II and LC2W) indicating very close genetic relatedness, which is also reflected in strain clustering based on overall gene content and MLST analysis (Figures 2 and 3). Several sets of contiguous spacers were also conserved between cluster F strains and strains Lc-10 and Zhang, which suggest common ancestry or HGT inheritance of this CRISPR locus. In contrast, spacer content showed more typical hypervariability across the other strains, with only 1 spacer shared between UW1 and UCD174 (Figure 5B). For Ldbu1, however, several sets of contiguous spacers were conserved between the dairy strains Lpc-37 and M36 (Figure 5B).

**Figure 6:**
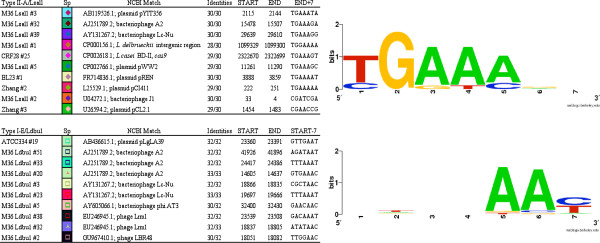
**CRISPR spacer homology to genetic elements and proto-spacer associated motifs. **Left, top 10 matches between CRISPR spacers and corresponding proto-spacers in phages, plasmids and chromosomal sequences. Numbers indicate the spacer position from the leader end. Levels of sequence similarity, location of the start and end of the match, and sequence immediately upstream (START-7) or immediately downstream (END+7) are provided. Right, conservation of certain nucleotides in the immediate vicinity of the proto-spacer sequence.

Analysis of spacer sequences revealed that numerous spacers show homology to *Lactobacillus* phages (Lc-Nu, A2, Lrm1, and J1) and plasmids (pYIT356, pREN, and pLgLA39) (Figure 6). Analysis of sequence conservation in the direct vicinity of proto-spacers that showed similarity or identity to CRISPR spacers revealed the presence of proto-spacer associated motifs (PAM) [65-67], namely, a conserved TGAAA immediately downstream of the Lsal1 protospacers, and AAY immediately upstream of the Ldbu1 protospacers (Figure 6). The TGAAA pentamer is homologous to the AGAAW PAM previously identified downstream of protospacers in *Streptococcus thermophilus* Type II systems [58,65,66].

Overall, CRISPR locus hypervariability across strains, in terms of occurrence, locus type and spacer content illustrates their functional value in response to environmental pressure, notably in providing resistance against phages. This polymorphism indicates CRISPR loci are desirable targets for typing of *L*. *casei* strains, in disagreement with a previous report [68]. The critical role that CRISPR-Cas systems play in resistance to viruses has been documented in dairy cultures [57,58,65,66,69], as well as environmental samples [60,70-74]. The occurrence of numerous *L*. *casei* CRISPR spacers with homology to *Lactobacillus* phages (notably Lc-Nu, Lrm1, A2 and phi AT3) that prey upon closely related species (Figure 6) combined with the larger numbers of spacer sequences in strains isolated from commercial cheese production environments (Lc-10, Lpc-37 and M36) further underscores the selective pressure against phage infection that exists in industrial dairy manufacturing environments. The propensity of hypervariable CRISPR loci for HGT [75] is also illustrated within *L*. *casei* by the co-occurrence of various IS elements for both types of loci (Figure 5A), the sharing of contiguous spacer sets across strains that belong to different phylogenetic clusters (Figures 2 and 5B), and the skewed GC content of *cas* genes (50-63% for Ldbu1 *cas* genes versus 46.5% genome-wide content). Overall, these results highlight the reliance of *L*. *casei* strains on CRISPR-Cas systems to provide immunity against invasive elements, as previously shown in bacteria and archaea [57,59,60,76,77].

### Evolution via genome decay

Our results and previous studies [21,28] have indicated HGT is a dominant force in genome evolution of *L*. *casei*, but a prior CGH experiment also provided evidence for a genetically distinct and geographically distributed cluster of *L*. *casei* cheese specialists whose evolution was accompanied by extensive decay of genes involved in carbohydrate utilization and transcriptional regulation [28]. This hypothesis is supported by the fact that energy production in *L*. *casei* is primarily derived from carbohydrate fermentation, so niche adaptation should be heavily predicated by the ability of strains to utilize available carbohydrate. Fermenting plant material, for example, can contain a diverse array of simple and complex carbohydrates as well as sugar alcohols [27], and many of these substrates will also be encountered in the GI tract as a consequence of diet. Thus, the ability to utilize C5 sugars and certain C5 and C6 sugar alcohols is more prevalent in *L*. *casei* isolated from plant material and the human GI tract than in cheese isolates [30]. The overlap in carbohydrate availability and use by *L*. *casei* associated with plants or the gastrointestinal tract also supports the hypothesis that many strains from these environments should be viewed as ecological generalists, whereas adaptation to cheese has been accompanied by extensive genome decay that, ultimately, resulted in niche specialization [28].

To explore the role of genome decay in the relationship between niche adaptation and substrate utilization, we tested a subset of nine stains (ATCC 334, 21/1, 32G, M36, CFR28, T71499, 12A, UW1 and UW4) distributed across the major MLST-defined *L*. *casei* lineages [28,30] for the ability to grow in CDAA supplemented with one of 60 different substrates associated with plant, gut, or dairy niches. Growth was detected on a total of 30 substrates, with individual strains able to utilize as few as 17 to as many as 26 different substrates (Additional file 5: Figure S3). Results showed the two cheese specialists UW1 and UW4, which share the same MLST lineage (Figure 3), had the most restricted substrate profile with growth on 18 and 17 different substrates, respectively. Each of the other strains was able to utilize at least 20 different carbohydrates, while two of the corn silage isolates (32G and 12A) each grew on 26 substrates, although their profiles were not identical (Additional file 5: Figure S3).

The genetic basis for utilization of many of these substrates is unknown, and efforts to detail the impact of genome decay on different substrate profiles is further challenged by the fact that most of *L*. *casei* genome sequences used for this study are incomplete. Nonetheless, evidence for genome decay in the evolution of the cheese specialist strains UW1 and UW4 was observed in regard to genes for inulin, sucrose and cellobiose utilization. In *L*. *casei*, the ability to ferment sucrose and inulin is encoded by an operon for fructooligosaccharide utilization (*fos*) [78,79] that is present in the other seven strains tested, which were all sucrose- and inulin-positive, but completely absent in UW1 and UW4.

**Figure 7:**
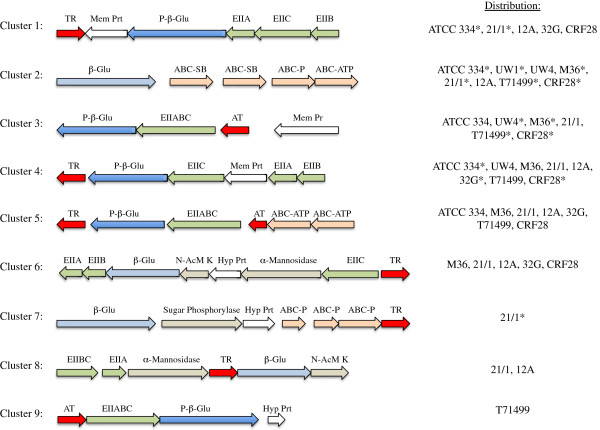
***L*. *casei *gene clusters that may function in cellobiose uptake and hydrolysis. **Asterisks identify clusters that are predicted to be nonfunctional in particular strains due to the presence of one or more pseudogenes. Abbreviations: P-β-Glu, phospho-β-glucosidase; β-Glu, β-glucosidase; ABC-ATP, ABC-P, and ABC-SB represent ATP-binding, periplasmic, and substrate-binding components, respectively, of an ABC transport system; EIIABC, EIIA, EIIB, EIIBC, or EIIC, represent sugar-specific enzymes for a PTS transport system; AT, antiterminator protein; TR, transcriptional regulator; Mem Prt, predicted membrane protein of unknown function; Hyp Prt, hypothetical protein; N-AcM K, N-acetymannosamine kinase. Locus tags in *L*. *casei *ATCC 334 for the glucosidase CDS in clusters 1–5 are LSEI_0448, LSEI_0700, LSEI_1104, LSEI_1778, and LSEI_2191, respectively. Locus tags in 21/1 for the glucosidase CDS in clusters 6–8 are LCA211_0376, LCA211_0004, and LCA211_2196, respectively. The locus tag in T71499 for the glucosidase CDS in cluster 9 is LCAT71499_1435.

In contrast to the single *fos* operon, we identified nine distinct gene clusters among the 17 *L*. *casei* genomes studied here that may function in cellobiose utilization (Figure 7). Cellobiose is a disaccharide formed by enzymatic or acid hydrolysis of cellulose that, like sucrose and inulin, may be encountered in plant material or in the human gastrointestinal tract but not in milk or cheese. Each of the nine strains analyzed in this part of the study possessed single copies of one (UW1) to eight (21/1 and 12A) of these gene clusters (Figure 7), and all but UW1 were able to ferment cellobiose (Additional file 5: Figure S3). While the function of each cluster in cellobiose utilization (as opposed to other β-glucosides) has not been demonstrated, data for strains ATCC 334 and UW4 show at least two of these clusters must enable cellobiose fermentation in *L*. *casei* (Figures 7 and S3). Cross comparison of the distribution of clusters 1 to 6 across the MLST lineages for the nine *L*. *casei* strains tested (Figures 3 and 7) provides clear evidence of genome decay; many clusters are entirely absent, presumably due to deletion events, and all but 12A contained at least one cluster that was predicted to be nonfunctional due to frameshift mutations (Figure 7). The single cluster in the cellobiose-negative strain UW1, for example, was predicted to be nonfunctional (Figure 7). Finally, examination of all 17 genomes included in this study confirmed the cheese specialists UW1 and UW4 had the fewest total cellobiose clusters, and were the only strains lacking cluster 5.

## Conclusions

In bacteria, the concept of species is traditionally anchored to features that are encoded by the core genome, but which often do not adequately describe the genetic diversity that is characteristic of a particular species [22,80]. Thus, there is increasing awareness that a large number of strains, preferably of disparate origin, must be sequenced to gain an accurate understanding of the evolution and biology of a particular bacterial species. To our knowledge, this is the first study to explore genome evolution and diversity in an ecologically flexible lactic acid bacterium using genome sequences from a large number of ecologically divergent strains.

As is depicted in Figure 4, the 17 *L*. *casei* genomes included in the study were not sufficient to capture the full pan-genome of this remarkable species. Indeed, mathematical extrapolation of these data indicated only 2/3 of the actual *L*. *casei* pan-genome has been sequenced to date (5,935 orthologous clusters found versus an estimated 9,072 total). Evidence for a much larger, open pan-genome was also provided by hierarchical clustering of strains based on overall gene content, since 2 of the 6 clusters (A and D) currently contain single isolates. Overall, our findings indicate *L*. *casei* has an open and distributed supragenome that is approximately 3.2 times larger than the average genome size for individual strains.

Characterization of the *L*. *casei* supragenome suggested its composition has been influenced by ecological co-localization with other bacterial species, especially lactobacilli. These findings provide additional confirmation that the DGH extends to non-pathogenic species [42], and indicate ecologically flexible bacteria like *L*. *casei* have access to a supragenome whose composition might be viewed as a subset of the microbial metagenome within a particular ecological niche. Finally, our results also provide support for the hypothesis that HGT has been a dominant force in adaptation of *L*. *casei* to new habitats and lifestyles, and that evolution of a genetically distinct and geographically distributed cluster of *L*. *casei* cheese specialists has been accompanied by extensive decay of genes associated with carbohydrate utilization [28].

## Methods

### Genome sequencing and assembly

Genomic DNA was extracted using a MasterPure Gram-Positive DNA Purification Kit (Epicentre Biotechnologies, Madison, WI). All 12 of the genome drafts were sequenced using a whole-genome shotgun strategy by pyrosequencing (GS FLX Titaninum 454 Life Sciences). The Lpc-37 and Lc-10 genomes were sequenced by University of Illinois-Urbana Champaign under contract services. The remaining 10 genomes were sequenced as a contract service by the Génome Québec Innovation Centre at McGill University (Montreal, Canada). Resulting sequences were assembled *de novo* using NGen SeqMan 2.0 (DNAstar, Madison, WI). The draft contigs for each genome assembly were compared to all five published genomes (Table 1) using progressive Mauve genome alignment software [81] and visually inspected for the best overall matching Local Collinear Blocks (LCBs). Based on the best matches, each draft was individually ordered and contigs oriented, but unlocated contigs were not scaffolded. Using the new contig orientation and order, the drafts assemblies were improved by hand curation *in silico* using SeqMan 8.1.2 (DNAstar). For strain Lpc-37, the draft genome assembly was also validated by comparison to a *NheI* optical restriction map (OpGen Inc, Gaithersburg MD) (see Additional file 1: Figure S1 in supplementary online material).

Initial annotations were generated using the RAST annotation server (http://rast.nmpdr.org/) with subsequent manual inspection and curation, including comparative analyses to improve consistency among orthologous groups. Pseudogene assignments were not exhaustive and were based on the available sequence; they may be influenced by sequencing artifacts due to the draft nature of the genomes.

### Ortholog predictions

Ortholog predictions were made based on reciprocal-best Basic Local Alignment Search Tool (BLAST; http://blast.ncbi.nlm.nih.gov/Blast.cgi) hits using a cutoff of 80% identity over 80% of the length. Confirmation of the reciprocal BLAST data and additional ortholog predictions were generated from whole-genome Mauve alignment data using a custom script and the A Systematic Annotation Package (ASAP) for Community Analysis of Genomes database (https://asap.ahabs.wisc.edu) [82]. This script uses synteny to determine whether features should be confirmed as orthologs, and can add orthologs based on genome context in cases where the BLAST results are ambiguous (e.g. paralogs) or that fail the cutoffs. This allows the initial BLAST assessment to be more strict, preventing the inclusion of homologs in the ortholog table and providing a one-to-one ortholog relationship between genomes.

### CDS and MLST dendrograms

A present/absent matrix of the CDS in all 17 strains of *L*. *casei* was created by a custom script and used to generate a dendrogram by the Ward method of hierarchical clustering (JMP version 9). Multiple sequence alignments were performed using molecular evolutionary genetic analysis (MEGA) software version 4 (http://www.megasoftware.net). A minimum evolution (ME) tree for all 17 *L*. *casei* strains was constructed using MEGA 4 based on the results of a bootstrapping test (1000 replicates) of strain phylogeny [83]. The phylogenetic tree was linearized assuming equal evolutionary rates in all lineages [84]. The evolutionary distances were computed using the modified Nei-Gojobori method [85] and are presented in the units of the number of synonymous substitutions per synonymous site. All positions containing gaps and missing data were eliminated from the dataset (complete deletion option).

To estimate the divergence time among different *L*. *casei* clusters, the concatenated sequences of 6 MLST loci (*ftsZ*, *metRS*, *mutL*, *pgm*, *nrdD*, and *polA*) were analyzed as described previously [30]. Divergence times between different clusters were indicated in a scale of millions of years. Calculations were based on the number of single nucleotide substitutions in each strain, and the estimated rate of single nucleotide substitutions between *Escherichia coli* and *Salmonella enterica* of 4.7 × 10^-9^ per site per year [86,87].

### Estimation of the *L*. *casei* core and pan-genome size

Protein coding features in each genome were grouped using OrthoMCL [88] with default settings and the resulting data were uploaded to the ASAP database. These data were retrieved from the database with a custom script that organized the feature IDs into a tab-delimited matrix allowing for identification of accessory genes (all features not included in the core genome) and strain-specific genes (unique to a single isolate) by inspection of this table. The best hits of the strain-specific features from a BLAST [89] analysis against the nr database using default parameters were collected and are presented in Additional file 2: Table S1. Graphical depictions of *L*. *casei* pan and core genome data, including the heat map, were generated using a custom php-based script that polls these data from the ASAP database and builds a present/absent matrix. Pan and core genome size estimates were derived with an R-package using a binomial mixture model [48].

### CRISPR identification and characterization

CRISPR loci were identified using a combination of homology to previously indentified repeats [61], *de novo* identification using CRISPRFinder [90] and repeat region identification using Dotter [91]. Spacers were visualized as previously described [66], and homologies to foreign genetic elements were assessed using BLAST [92]. CRISPR loci sequences were independently confirmed by Sanger sequencing of PCR amplicons. Nucleotide conservation between CRISPR spacers and corresponding proto-spacers in phages, plasmids and chromosomal sequences were visualized using WebLogo [93].

### Carbohydrate utilization

*L*. *casei* strains 21/1, 12A, M36, UCD174, A2-362, 32G, T71499, CRF28, UW1, UW4, and ATCC 334 were transferred from -80C freezer stocks into MRS broth (Difco Laboratories, Detroit, MI) and grown overnight (16–18 h) at 37°C. Strains were inoculated at 1% (v/v) into a filter-sterilized chemically defined amino acid medium (CDAA) with 25 mM galactose and incubated 16–18 h at 37°C. The CDAA was comprised of 114 mg sodium acetate, 171 mg sodium citrate, 171 mg ammonium chloride, 343 mg potassium phosphate (monobasic), 343 mg potassium phosphate (dibasic), 114 mg magnesium sulfate tetrahydrate, 6 mg iron sulfate hexahydrate, 6 mg manganese sulfate tetrahydrate, 4 g sodium chloride, 228 mg L-phenylalanine, 455 mg L-tyrosine, 6 mg L-adenine, 6 mg L-guanine, 6 mg L-uracil, 6 mg L-xanthine, 351 mg DL-aspartate, 245 mg L-glutamate, 545 mg L-tryptophan, 443 mg L-alanine, 312 mg L-arginine, 746 mg L-asparagine, 857 mg L-cysteine, 816 mg L-glutamine, 341 mg glycine, 900 mg L-histidine, 923 mg L-isoleucine, 326 mg L-leucine, 428 mg L-lysine, 148 mg DL-methionine, 93 mg L-proline, 946 mg DL-serine, 404 mg DL-threonine, 651 mg L-valine, 24 mg L-cystine, 1 mL trace elements solution [94], 0.4 mL Tween 80, 0.4 mL Tween 20, 0.4 mL Tween 60, plus 10 mL RPMI 1640 vitamin solution (100X; added prior to experimentation), and pH adjusted to 5.5.

Cells were collected by centrifugation at 13,000 x g for 5 min at 4°C, then suspended in CDAA lacking carbohydrate. Samples of each strain were then added to a final absorbance at 595nm (A_595_) of 0.1 into 1 mL CDAA adjusted to pH 5.5 that contained 2 mM galactose as a growth booster plus one of the following substrates: 25 mM meso-erythritol, D-xylose, D-ribose, D-arabinose, D-adonitol, D-arabitol, D-xylitol, D-glucose, D-mannose, D-galactose, D-fructose, lactone, D-mannitol, D-galactitol, D-sorbitol, myo-inositol, D-glucosamine, n-acetyl D-glucosamine, sialic acid (Indofine Chemical Company, Inc., Hillsborough, NJ), lactulose, D-lactose, D-sucrose, D-turanose, D-maltose, D-cellobiose, D-trehalose, D-maltitol, D-lactitol, D-raffinose, or D-melezitose; or 4.5 mg/mL (which is equivalent to 25 mM glucose) of heparin, N-acetyl-D-galactosamine, fucose, panose, galactosamine, amylopectin, high methylated pectin, stachyose, pectin, arabinogalactan, rhamnose, inulin, mucin, or phytic acid; or 12.5 mg/mL isomaltose, galacturonic acid, polydextrose, glucuronic acid, amygdalin, maltotriose, pullulan, amylopectin, carboxymethyl cellulose, xylan, lignin, α-cyclodextrin, β-cyclodextrin, γ-cyclodextrin, dextrin, or amylose. Unless noted, substrates were purchased from Sigma-Aldrich Co. (St. Louis, MO). Strains A2-362 and UCD174 were not included in these studies because they were unable to grow well in CDAA.

Inoculated mixtures were incubated at 37°C under static conditions. Aliquots (50 μl) were periodically collected over a 48 h period, placed in a 96-well microtiter plate, and A_595_ was determined using a 96 well plate-reader (Bio-Rad, Hercules, CA). Uninnoculated mixtures containing individual substrates were used as blanks for the plate reader. Carbohydrates that produced a turbid sample at time 0 (amylopectin, mucin, carboxymethyl cellulose, xylan, lignin, α-cyclodextrin, γ-cyclodextrin, β-cyclodextrin, dextrin, and amylose) were diluted in a 0.9% sterile saline solution and plated on MRS agar (Difco Laboratories) using the drop plate method [95]. Plates were incubated at 37°C for 48 h prior to colony enumeration.

All growth experiments were performed in triplicate, and the ability to utilize a particular substrate was determined by two-tailed student’s t-test comparison (α = 0.05) between the mean A_595_ values from cells incubated in CDAA containing no carbohydrate versus CDAA containing the substrate of interest. A dendrogram showing the relationships between strains in regard to substrate utilization was created using the Ward method in hierarchical clustering (JMP version 9, SAS Institute Inc., Cary, NC).

### Identification of cellobiose gene clusters

Putative gene clusters for cellobiose utilization were identified by keyword screening of gene annotations for “β-glucosidase”, and by BLAST homology searches of each genome using β-glucosidase protein sequences from the Carbohydrate-Active enZYmes database (http://www.cazy.org). Neighboring genes were then examined for support functions such as cellobiose transport and transcription activators or terminators.

## Abbreviations

BLAST: Basic local alignment search tool; CDS: Coding sequence; CGH: Comparative genome hybridization; CRISPR: Clustered regularly interspaced short palindromic repeats; DGH: Distributed genome hypothesis; HGT: Horizontal gene transfer; LCB: Locally collinear blocks; MLST: Multi-locus sequence typing; OD: Optical density; PAM: Proto-spacer associated motifs.

## Competing interests

BS, WM, PH, RB, and JLS received salary and/or funding from DuPont, a supplier of bacterial cultures to the food industry. Peggy Steele, a member of JLS’s family, is also employed by DuPont.

## Authors’ contributions

JRB, JLS, RB, BS, ECN-E, NP, HC, and PH conceived and designed the study; JRB, ECN-E, BS, KT, JH, WM, and RB performed experiments and bioinformatic analyses; JRB, JLS, KT, JH, ECN-E, HC, RB, BS, NP, and WM contributed to data interpretation; PH contributed reagents, materials and analytical/technical expertise; JRB, JLS, ECN-E, RB, and BS wrote the paper. All authors read and approved the final manuscript.

## Supplementary Material

Additional file 1 **Figure S1. **Comparative analysis of the *L*. *casei *Lpc-37 chromosome Optical Map with its *in silico *equivalent. Shown are: (a) *in silico *Optical Map contigs were ordered in an alternating pattern to demonstrate the boundaries of the current ordered draft, without unplaced contigs; (b) The *Nhe*I Optical Map of Lpc-37 used as a reference to independently validate the assembly and order of the contig draft. Orange shaded regions indicate where alignment match more than once and blue regions indicate a single alignment match. Green boxes highlight the regions that have no coverage in the ordered draft. Site 1 is approximately 30kb, site2 is approximately 70kb and site 3 is approximately 10kb. Part (c) depicts the concatenated sequence of the ordered contigs in the draft of Lpc-37, without the unplaced contigs; and (d) shows the remaining unplaced contigs were very small and could not be ordered based on Optical Map alignment. Click here for file

Additional file 2 **Table S1. **Protein homology data for predicted strain-specific accessory gene products in *L. casei *strains. Click here for file

Additional file 3 **Table S2.** Evidence for recent intraspecific and niche-associated horizontal gene transfer in *L*. *casei*. Click here for file

Additional file 4 **Figure S2. **Polycistronic clusters that may reflect recent intraspecific and niche-associated horizontal gene transfer in *L. casei*. Panel A, graphic representation of an ABC sugar transport system found in *L. casei *UW1 and *L. brevis *ATCC 367. Abbreviations: *lacL *= β-galactosidase large subunit; *lacM* = β-galactosidase small subunit; *LVIS*_*2257 *= ABC-type sugar transport system, ATPase component; *LVIS*_*2256 *= multiple sugar ABC transporter, substrate-binding protein; *LVIS*_*2254 *and *LVIS*_*2255 *= multiple sugar ABC transporter, membrane-spanning permease protein; *LVIS*_*2252 *= oxidoreductase; *reg *= transcriptional regulator; hyp = hypothetical protein; *tn *= transposase. Panel B, graphic representation of the plasmid-coded partial *lar *operon present in *L*. *casei *BD-II and its alignment with a corresponding region of the *L*. *plantarum *genome. Abbreviations: *cbiM *= cobalt ABC transporter, substrate-binding protein; *lp*_*0103 *= transcriptional regulator; *larA* = phosphoribosylcarboxy-aminoimidazole (NCAIR) mutase; *larB *= unknown function; *larC1* and *larC2 *= unknown function; *glpF1 *= glycerol uptake facilitator protein; *larE *= unknown function; *lp*_*0111 *= quinone oxidoreductase; *thiM *= hydroxyethylthiazole kinase. Panel C, graphic representation of the tartrate dehydratase operon and flanking genes found in *L*. *casei* UCD174 and their alignment with a corresponding region in the *L*. *plantarum *genome. Abbreviations: *mtsA *= manganese transport system, ATP binding protein; *citG *= 2'-(5''-triphosphoribosyl)-3-dephospho-CoA synthase; *ttdR *= transcription regulator tartrate operon; *ttdA *= tartrate dehydratase α subunit; *ttdB *= tartrate dehydratase β subunit; *ttdP *= 2-oxoglutarate /malate translocator protein; *aroB *= 3-dehydroquinate synthetase; *aroA * = 3-deoxy-7-phosphoheptulonase synthase; *aroD1 *= shikimate 5-dehydrogenase; *tkt2* = transketolase. Vertical lines in each panel denote the region that displays 98-99% nucleotide sequence identity between each paired comparison (see text). Click here for file

Additional file 5 **Figure S3. **Variable growth among nine *L. casei* strains on selected substrates. Blue indicates growth and yellow indicates negligible growth within 48 hours. Hierarchical clustering of strains based on substrate utilization profile is also depicted. Carbohydrates which were utilized for growth by all strains tested include D-glucose (shown), as well as D-mannose, D-galactose, D-fructose, D-mannitol, D-turanose, and D-melezitose galactosamine (not depicted). Growth was not observed with meso-erythritol, D-arabinose, D-adonitol, D-arabitol, D-xylitol, D-galactitol, myo-inositol, sialic acid, heparin, fucose, amylopectin, rhamnosemucin, phytic acid, galacturonic acid, glucuronic acid, amylopectin, carboxymethyl cellulose, xylan, lignin, α-cyclodextrin, β-cyclodextrin, γ-cyclodextrin, dextrin, and amylose. Click here for file
