# Supplementary figures and images for: Analysis of the Lactobacillus casei supragenome and its influence in species evolution and lifestyle adaptation
Source: BMC Genomics. 2012 Oct 5;13:533. doi: 10.1186/1471-2164-13-533 (PMC3496567; doi:10.1186/1471-2164-13-533)

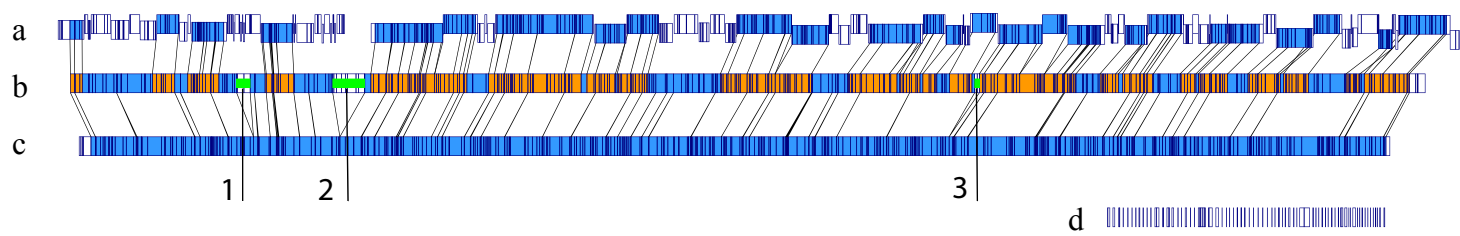

Supplement: Additional file 1 — Figure S1. Comparative analysis of the L. casei Lpc-37 chromosome Optical Map with its in silico equivalent. Shown are: (a) in silico Optical Map contigs were ordered in an alternating pattern to demonstrate the boundaries of the current ordered draft, without unplaced contigs; (b) The NheI Optical Map of Lpc-37 used as a reference to independently validate the assembly and order of the contig draft. Orange shaded regions indicate where alignment match more than once and blue regions indicate a single alignment match. Green boxes highlight the regions that have no coverage in the ordered draft. Site 1 is approximately 30kb, site2 is approximately 70kb and site 3 is approximately 10kb. Part (c) depicts the concatenated sequence of the ordered contigs in the draft of Lpc-37, without the unplaced contigs; and (d) shows the remaining unplaced contigs were very small and could not be ordered based on Optical Map alignment. [file 1471-2164-13-533-S1.pdf]

**A.** *L. brevis* ATCC 367:

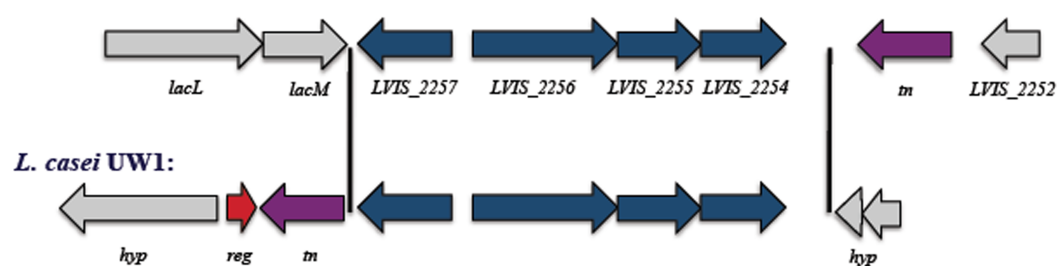

**B.** *L. plantarum*:

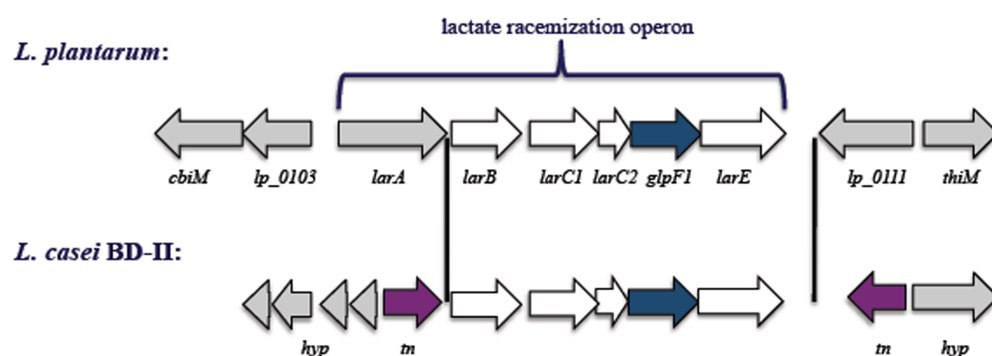

**C.** *L. plantarum*:

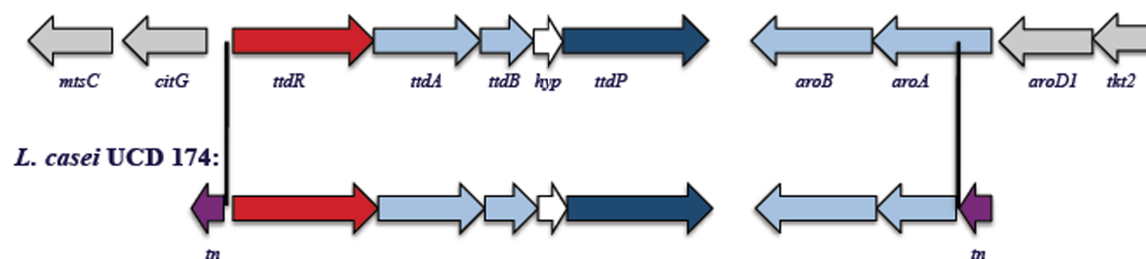

Supplement: Additional file 4 — Figure S2. Polycistronic clusters that may reflect recent intraspecific and niche-associated horizontal gene transfer in L. casei. Panel A, graphic representation of an ABC sugar transport system found in L. casei UW1 and L. brevis ATCC 367. Abbreviations: lacL = β-galactosidase large subunit; lacM = β-galactosidase small subunit; LVIS_2257 = ABC-type sugar transport system, ATPase component; LVIS_2256 = multiple sugar ABC transporter, substrate-binding protein; LVIS_2254 and LVIS_2255 = multiple sugar ABC transporter, membrane-spanning permease protein; LVIS_2252 = oxidoreductase; reg = transcriptional regulator; hyp = hypothetical protein; tn = transposase. Panel B, graphic representation of the plasmid-coded partial lar operon present in L. casei BD-II and its alignment with a corresponding region of the L. plantarum genome. Abbreviations: cbiM = cobalt ABC transporter, substrate-binding protein; lp_0103 = transcriptional regulator; larA = phosphoribosylcarboxy-aminoimidazole (NCAIR) mutase; larB = unknown function; larC1 and larC2 = unknown function; glpF1 = glycerol uptake facilitator protein; larE = unknown function; lp_0111 = quinone oxidoreductase; thiM = hydroxyethylthiazole kinase. Panel C, graphic representation of the tartrate dehydratase operon and flanking genes found in L. casei UCD174 and their alignment with a corresponding region in the L. plantarum genome. Abbreviations: mtsA = manganese transport system, ATP binding protein; citG = 2'-(5''-triphosphoribosyl)-3-dephospho-CoA synthase; ttdR = transcription regulator tartrate operon; ttdA = tartrate dehydratase α subunit; ttdB = tartrate dehydratase β subunit; ttdP = 2-oxoglutarate /malate translocator protein; aroB = 3-dehydroquinate synthetase; aroA = 3-deoxy-7-phosphoheptulonase synthase; aroD1 = shikimate 5-dehydrogenase; tkt2 = transketolase. Vertical lines in each panel denote the region that displays 98-99% nucleotide sequence identity between each paired comparison (see text). [file 1471-2164-13-533-S4.pdf]

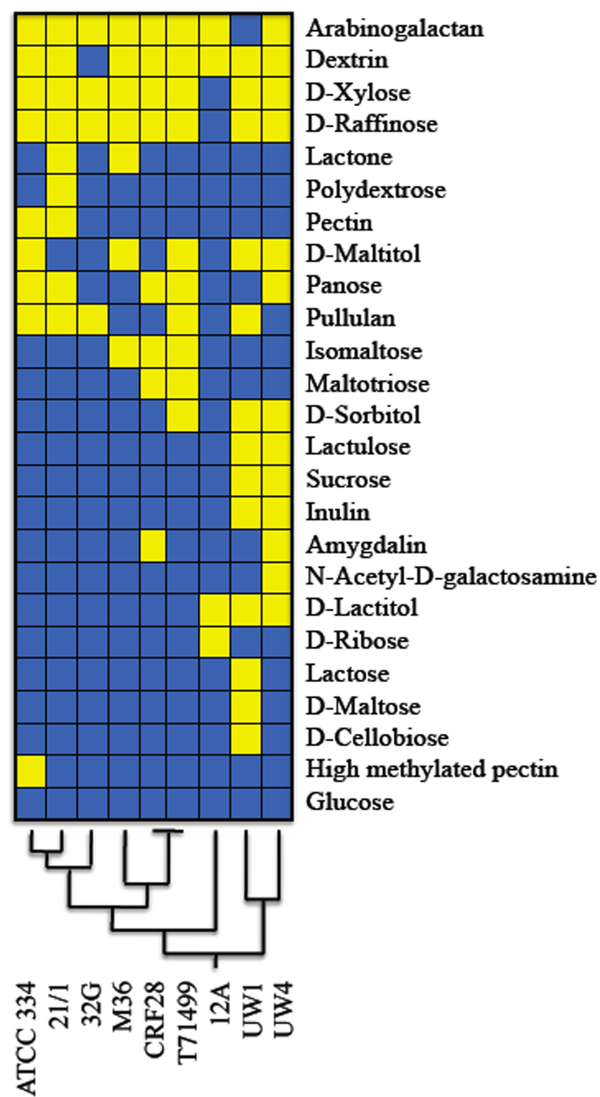

Supplement: Additional file 5 — Figure S3. Variable growth among nine L. casei strains on selected substrates. Blue indicates growth and yellow indicates negligible growth within 48 hours. Hierarchical clustering of strains based on substrate utilization profile is also depicted. Carbohydrates which were utilized for growth by all strains tested include D-glucose (shown), as well as D-mannose, D-galactose, D-fructose, D-mannitol, D-turanose, and D-melezitose galactosamine (not depicted). Growth was not observed with meso-erythritol, D-arabinose, D-adonitol, D-arabitol, D-xylitol, D-galactitol, myo-inositol, sialic acid, heparin, fucose, amylopectin, rhamnosemucin, phytic acid, galacturonic acid, glucuronic acid, amylopectin, carboxymethyl cellulose, xylan, lignin, α-cyclodextrin, β-cyclodextrin, γ-cyclodextrin, dextrin, and amylose. [file 1471-2164-13-533-S5.pdf]
